# Induction of the Viable-but-Nonculturable State in *Salmonella* Contaminating Dried Fruit

**DOI:** 10.1128/AEM.01733-21

**Published:** 2022-01-25

**Authors:** Victor Jayeola, J. M. Farber, S. Kathariou

**Affiliations:** a Department of Plant and Microbial Biology, North Carolina State University, Raleigh, North Carolina, USA; b Department of Food Science, University of Guelph, Guelph, Ontario, Canada; c Department of Food, Bioprocessing and Nutrition Sciences, North Carolina State University, Raleigh, North Carolina, USA; The Pennsylvania State University

**Keywords:** dried fruit, survival, low-moisture foods, *Salmonella*, confocal microscopy, viable but nonculturable

## Abstract

Salmonella can become viable but nonculturable (VBNC) in response to environmental stressors, but the induction of the VBNC state in Salmonella contaminating ready-to-eat dried fruit is poorly characterized. Dried apples, strawberries, and raisins were mixed with a five-strain cocktail of Salmonella at 4% volume per weight of dried fruit at 10^9^ CFU/g. The inoculated dried fruit were then dried in desiccators at 25°C until the water activity (*a*_w_) approximated that of the uninoculated dried fruit. However, Salmonella could not be recovered after drying, not even after enrichment, suggesting a population reduction of approximately 8 log CFU/g. To assess the potential impact of storage temperature on survival, dried apples were spot-inoculated with the Salmonella cocktail, dried under ambient atmosphere at 25°C, and stored at 4 and 25°C. Spot inoculation permitted recovery of Salmonella on dried apple after drying, with the population of Salmonella decreasing progressively on dried apples stored at 25°C until it was undetectable after about 46 days, even following enrichment. The population decline was noticeably slower at 4°C, with Salmonella being detected until 82 days. However, fluorescence microscopy and laser scanning confocal microscopy with the LIVE/DEAD *Bac*Light bacterial viability system at time points at which no Salmonella could be recovered on growth media even following enrichment showed that a large proportion (56 to 85%) of the Salmonella cells on the dried fruit were viable. The data suggest that the unique combination of stressors in dried fruit can induce large numbers of VBNC cells of Salmonella.

**IMPORTANCE** Salmonella is a leading foodborne pathogen globally causing numerous outbreaks of foodborne illnesses and remains the leading contributor to deaths attributed to foodborne disease in the United States and other industrialized nations. Therefore, efficient detection methods for Salmonella contaminating food are critical for public health and food safety. Culture-based microbiological methods are considered the gold standard for the detection and enumeration of Salmonella in food. Findings from this study suggest that unique stressors on dried fruit can induce the VBNC state in Salmonella, thus rendering it undetectable with culture-based methods even though the bacteria remain viable. Therefore, strong consideration should be given to using, in addition to culture-based methods, microscopic and molecular methods for the accurate detection of all viable and/or culturable cells of Salmonella contaminating dried fruit, as all of these cells have the potential to cause human illness.

## INTRODUCTION

Bacteria in the viable-but-nonculturable (VBNC) state fail to grow under laboratory conditions, which would normally permit growth, although they remain alive and metabolically active (1). Since the pioneering studies on the induction of the VBNC state in Escherichia coli and Vibrio cholerae (2, 3), many foodborne pathogens have been reported to enter into the VBNC state in response to stressful environmental conditions (4–6). Various stressors including desiccation, low pH, UV-radiation, and thermosonication have been shown to induce the VBNC state in diverse serotypes of Salmonella (7–10).

Significant attention has been paid to the potential for stressors commonly encountered in foods or food processing environments to induce the VBNC state (11). A large percentage of the population of Salmonella enterica serovar Thompson contaminating spinach leaves transitioned into the VBNC state when exposed to chlorine (12). Treatment of chicken breasts with lactic acid and peracetic acid induced the VBNC state in Salmonella contaminating the product (13). Moreover, Salmonella inoculated into grape juice became nonculturable but remained viable after storage at 4°C for 48 h (14). However, there are crucial knowledge gaps regarding the occurrence or induction of the VBNC state in Salmonella contaminating low-moisture foods (LMFs), defined as those with water activity (*a*_w_) below 0.85 (15).

Salmonella is a leading cause of foodborne illnesses globally (16). In the United States alone, Salmonella is estimated to cause 1 million cases of foodborne illnesses annually, accounting for 28% of all hospitalizations and 35% of all deaths attributed to known foodborne bacterial pathogens (17, 18). The U.S. Department of Agriculture Economic Research Service (USDA-ERS) estimated the average cost of foodborne illness from Salmonella in 2018 to be $4.1 billion (19). Contaminated LMFs are important vehicles for Salmonella outbreaks, accounting for 21% of investigated Salmonella outbreaks reported by the Centers for Disease Control and Prevention (CDC) from 2007 to 2018 (20). Worldwide, LMFs were involved in numerous outbreaks, with Salmonella responsible for 53% of these outbreaks (21), while an overwhelming majority (>83%) of foodborne outbreaks associated with LMFs in the United States between 2007 and 2018 involved Salmonella (20).

Several studies have shown that Salmonella is able to survive low-moisture conditions in LMFs and dry abiotic surfaces for an extended time period (15, 22). It is conceivable that VBNC Salmonella may have contributed to LMF-associated outbreaks where low numbers of Salmonella cells were recovered from the implicated products (8, 23–25). However, experimental evidence for the induction of the VBNC state in Salmonella contaminating LMFs is lacking.

Unlike many other LMFs, dried fruit are characterized by a unique combination of traits, including low water activity (<0.6), high sugar content (38 to 66%), low pH (<4.5), and antimicrobial phenolic compounds (26–28). The survival of Salmonella on dried fruit is considerably lower than that on other LMFs (15, 22, 29). Nonetheless, Salmonella could still be recovered from certain dried fruit for weeks or months, especially at lower temperature (28), suggesting that the presence of Salmonella on dried fruit raises food safety and public health concerns. Furthermore, the extent to which fruit may harbor Salmonella in the VBNC state, which would not be detected by traditional culture-based approaches, remains unknown.

In the course of an investigation of the transcriptional responses of Salmonella contaminating various types of LMFs, we noted that transcripts could be readily obtained from Salmonella inoculated on dried apples at time points that failed to yield Salmonella on selective or nonselective media (30). Since bacterial RNA is generally short-lived, with a half-life ranging from seconds to only a few hours (31, 32), we hypothesized that Salmonella cells contaminating the dried apples may have transitioned into the VBNC state and, hence, became unculturable on growth media. Therefore, the objective of the current study was to assess the induction of the VBNC state in Salmonella contaminating dried fruit.

## RESULTS

Dried apples, dried strawberries, and raisins were directly provided by industry sources (hereafter referred to as “industry-supplied”), while Red Delicious (RD) and Granny Smith (GS) apples were purchased from local grocery stores and dried in the laboratory as described in Materials and Methods. The total aerobic bacterial count of industry-supplied dried apples and strawberries was below the limit of detection of 20 CFU/g, while raisins had 2 to 3 log CFU/g (data not shown). Similar numbers of aerobic bacteria (2 to 3 log CFU/g) were obtained from laboratory-dried Red Delicious and Granny Smith apples (data not shown). Salmonella was not detected in any of the dried fruit before inoculation. The average *a*_w_ of uninoculated dried fruit ranged from 0.41 (industry-supplied dried apples) to 0.64 (laboratory-dried Red Delicious apple) (Table 1), and the *a*_w_ of the inoculated dried fruit did not change significantly during storage (data not shown). The average pH of dried fruit homogenates ranged from 3.1 (dried Granny Smith) to 4.6 (raisins), and the Brix (the amount of dissolved solids in rinsates of dried fruit) ranged from 14 (laboratory-dried Red Delicious apple) to 29.4 degrees Brix (°Bx) (industry-supplied dried apples) as shown in Table 1.

**TABLE 1:** Water activity, pH, and Brix of dried fruit used in this study

Dried fruit	Water activity (*a*_w_)^*a*^			pH^*b*^	Brix (°Bx)^*b*^
	Uninoculated	After inoculation	After drying		
Industry-supplied dried apples	0.56	0.65	0.53	4.5	29.4
Raisins	0.42	0.77	0.44	4.6	21.4
Strawberries	0.41	0.63	0.49	4.4	24.1
Red Delicious apples	0.64	0.72	0.65	4.1	14
Granny Smith apples	0.51	0.64	0.51	3.2	17

a For *a*_w_ measurements, dried strawberries were sliced into smaller pieces while dried apple fragments and raisins were analyzed without size reduction as described in Materials and Methods. Water activity measurements were taken in triplicate using a benchtop water activity meter.

b To determine the pH and Brix, homogenates of dried fruit were prepared as described in Materials and Methods. The pH and the Brix of the homogenates were analyzed using a benchtop pH meter and a pocket refractometer, respectively, as described in Materials and Methods.

### The viable but nonculturable state was induced in Salmonella contaminating dried fruit.

**FIG 1:**
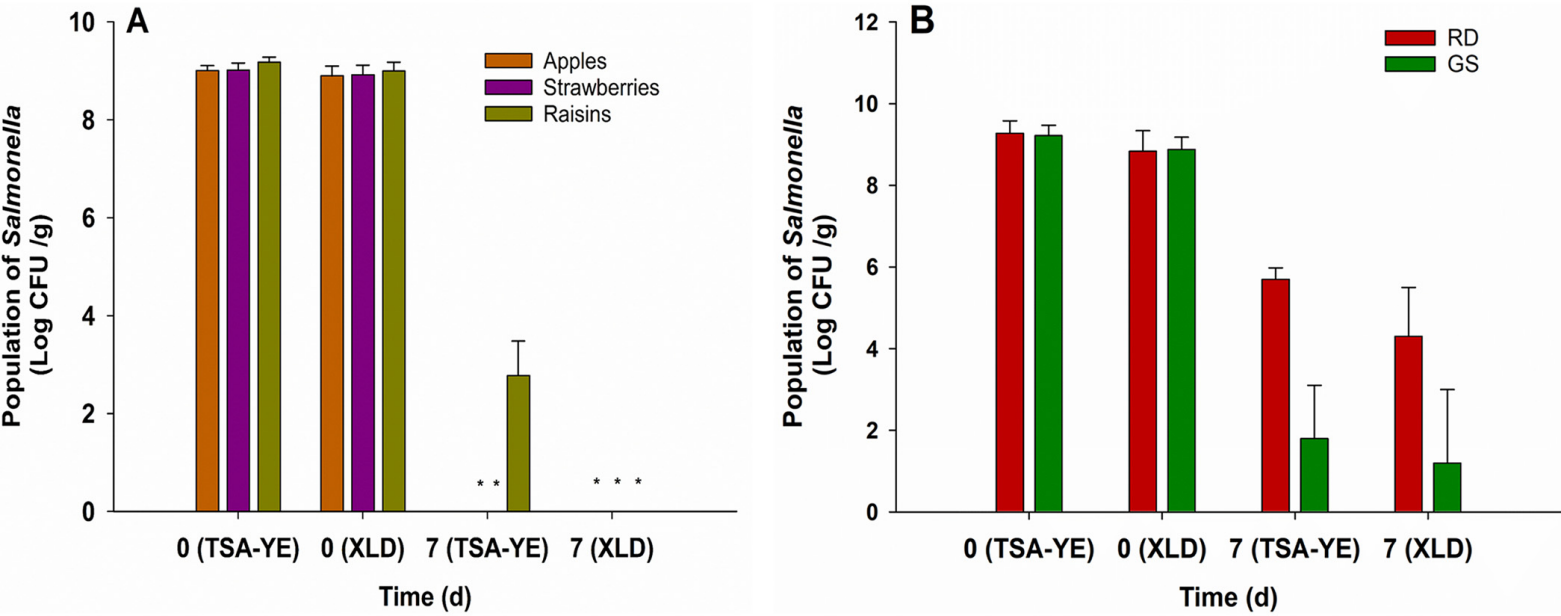
Survival of Salmonella on dried strawberries, raisins, and dried apples (A) and Red Delicious (RD) and Granny Smith (GS) apples (B). Apples, strawberries, and raisins were industry supplied, while Granny Smith (GS) and Red Delicious (RD) were purchased at retail and dried at the laboratory as described in Materials and Methods. The dried fruit were inoculated, dried, and stored as described in Materials and Methods. The population of Salmonella on the dried fruit was enumerated on nonselective (TSA-YE) and selective (XLD) media immediately after inoculation (T0) and after drying (T7) as described in Materials and Methods. Data represent averages from three independent trials with two samples processed per trial, and error bars indicate standard deviation. Salmonella was undetected on the dried Granny Smith apples in the first trial; thus, survival data represent average of the second and third trials. Black and red bars indicate counts on TSA-YE and XLD, respectively. *, Salmonella was below the limit of detection (20 cells/g).

Dried strawberries, apples, and raisins were inoculated in bulk with Salmonella by mixing the dried products with a Salmonella strain cocktail cell suspension to approximately 9 ± 0.2 log CFU/g. However, Salmonella could not be recovered on growth media (limit of detection [LOD], 20 CFU/g) after drying the fruits for 7 days to the original *a*_w_, even following enrichment (Fig. 1). Survival studies were also done with bulk-inoculated Red Delicious and Granny Smith apples dried in the laboratory in order to determine if the rapid loss in culturability was peculiar to the industry-supplied dried fruit, e.g., due to the possible addition of sulfites. After drying for 7 days, the levels of Salmonella on dried Red Delicious apples decreased from 9.2 CFU/g to 5.7 and 4.3 log CFU/g on Trypticase soy broth (TSB) containing 0.6% yeast extract (TSB-YE) supplemented with 1.2% agar (TSA-YE) and xylose lysine deoxycholate (XLD), respectively (Fig. 1). Even more pronounced reductions were seen with Granny Smith, where after drying for 7 days to the original *a*_w_, Salmonella was not detected in one trial, and populations were reduced to approximately 1.6 and 1.2 log CFU/g on TSA-YE and XLD, respectively, in the two other trials (Fig. 1).

Previous studies suggested that preadaptation of Salmonella to acidic pH via growth on glucose-supplemented TSA-YE improved its survival on dried fruit (28). To test the potential impact of low-pH preadaptation on the survival of Salmonella on dried apples, the Salmonella inoculum was prepared with cultures grown on TSA-YE supplemented with 1% glucose (TSA-YEG) (37°C, 24 h) to a final pH of 4.4, and then was bulk-inoculated on dried apples at 9.4 log CFU/g. However, the preadapted Salmonella cells also became undetectable after the fruit was dried for 7 days (<20 CFU/g), with no colonies observed on XLD or TSA-YE aerobically, anaerobically, or under microaerobic conditions (37°C, 24 h).

**FIG 2:**
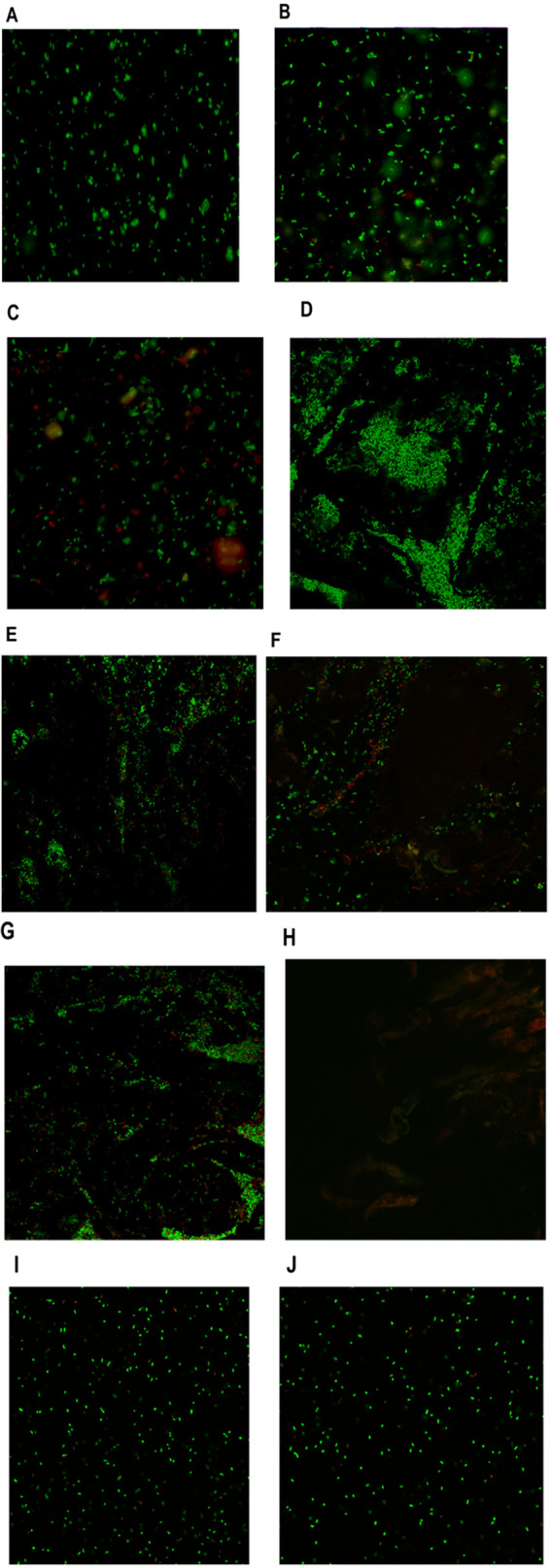
Visualization of live and dead Salmonella cells from inoculated dried fruit by fluorescence (A to C) or laser scanning confocal (D to J) microscopy. Live (green) and dead (red) cells were visualized after staining with the LIVE/DEAD BacLight bacterial viability kit as described in Materials and Methods. Inoculum suspensions (A), rinsate of industry-supplied, bulk-inoculated dried apples after drying for 7 days (B), rinsate of bulk-inoculated dried strawberries after drying for 7 days (C), surface of spot-inoculated dried apples after drying for 3 h (D), surface of spot-inoculated dried apples after storage for 50 days at 25°C (E), surface of spot-inoculated dried apples after storage for 110 days at 25°C (F), surface of spot-inoculated dried apples after storage for 110 days at 4°C (G), surface of uninoculated dried apples (H), inoculated apple juice with 30% fructose stored at 25°C for 5 days (I), and inoculated apple juice without fructose stored at 25°C for 5 days (J). Representative fields are shown. The red and green hues in panel H (uninoculated dried apples) were autofluorescence from the fruits.

Viability staining of rinsates of dried fruit, followed by visualization with fluorescence microscopy, revealed numerous Salmonella cells that were viable despite the absence of colonies on agar plates. In comparison to the inoculum where 99.5% of the cells fluoresced green (Fig. 2A), a significant fraction, i.e., 85 ± 9 and 67.3 ± 6%, of the rinsates of bulk-inoculated industry-supplied apples and strawberries, respectively, appeared to contain viable cells of salmonellae upon completion of the 7-day drying period (Fig. 2B and C). Since no fluorescing bacteria were detected on uninoculated dried apples (Fig. 2H) or strawberries (data not shown), the fluorescing bacterial cells on the inoculated products were considered to be Salmonella. Uninoculated raisins had significant background fluorescence from bacterial cells that were morphologically similar to Salmonella (data not shown) and, therefore, were not pursued further with fluorescence microscopy.

### Salmonella survived longer on spot-inoculated dried apples, with survival markedly enhanced at 4°C.

**FIG 3:**
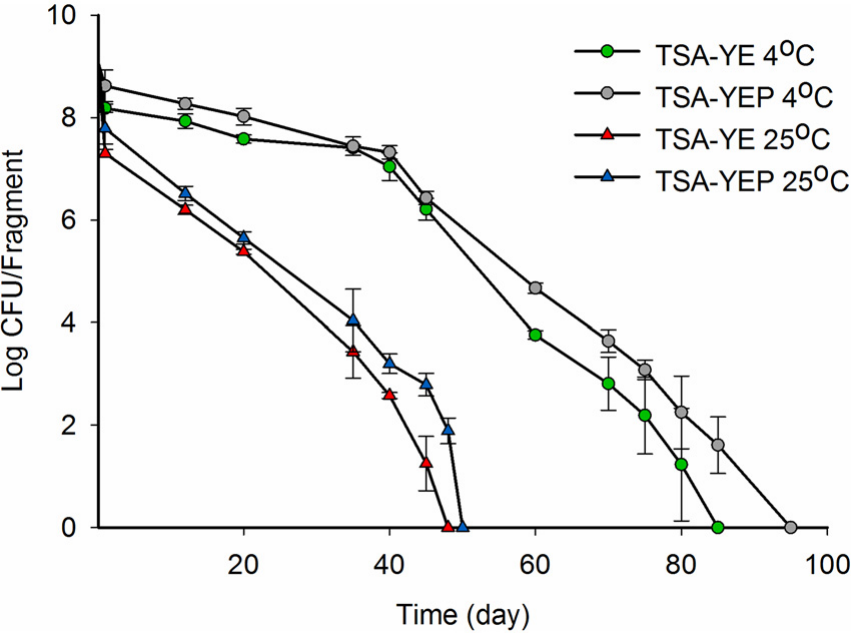
Survival of Salmonella on industry-supplied, spot-inoculated dried apples. Individual fragments of dried apples were inoculated, dried, and stored at 4 and 25°C as described in Materials and Methods. The population of Salmonella was enumerated on TSA-YE and TSA-YE supplemented with pyruvate (TSA-YEP) as described in Materials and Methods immediately after inoculation, after drying, and periodically during storage. Data represent averages from two independent trials with three samples processed per trial, and error bars indicate standard deviation.

The *a*_w_ of spot-inoculated (9.2 log CFU/fragment) dried apples immediately after inoculation and after drying was 0.75 and 0.54, respectively. In contrast to the rapid population reductions observed on bulk-inoculated dried apples (fruit homogeneously mixed with inoculum and dried in desiccators at 25°C) where no Salmonella could be recovered upon drying of the fruit to the original *a*_w_, there was no significant decline in the levels of Salmonella on the spot-inoculated apples (9.1 log CFU/fragment) after 3 h of drying under ambient conditions at 25°C. However, the populations decreased progressively upon storage at 25°C until Salmonella became nondetectable (<10 CFU/g) on TSA-YE plates on day 46 ± 2 (Fig. 3). Growth was also examined on pyruvate-supplemented media (TSA-YEP) to test if protection against oxidative stress would promote the resuscitation of VBNC Salmonella. Significantly longer maintenance of culturability (*P *< 0.0001) was noted when the spot-inoculated apples were stored at 4°C, where Salmonella could be detected for as long as 82 ± 3 days (Fig. 3). Furthermore, higher numbers of Salmonella tended to be recovered on TSA-YEP than on TSA-YE, regardless of the storage temperature, until Salmonella failed to grow on either medium (Fig. 3).

Even though Salmonella on spot-inoculated dried apples stored at 4 and 25°C failed to form colonies on either TSYE or TSA-YEP after 82 ± 3 and 46 ± 2 days, respectively (Fig. 3), confocal microscopy suggested a high percentage of viable Salmonella even after these time periods, e.g., 50 days at 25°C or 110 days at 4°C. The confocal microscopy data suggested that an estimated 68 ± 6% of the Salmonella population on spot-inoculated dried apples stored for 50 days at 25°C was viable (Fig. 2E), with the percentage decreasing significantly (*P *< 0.05) to 54 ± 4% at 110 days (Fig. 2F). Similarly, spot-inoculated dried apples stored for 110 days at 4°C contained 56 ± 9% of viable Salmonella, even though colonies were no longer obtained on growth media (Fig. 2G).

To estimate the total population of live cells on the dried apples, quantitative PCR (qPCR) was first used to determine the total (dead and live) populations of Salmonella on spot-inoculated industry-supplied dried apples stored for 50 and 110 days at 25 and 4°C, respectively. The concentration of genomic DNA (gDNA) isolated per fragment of inoculated apple after 3 h of drying, 50 days at 25°C, or 110 days at 4°C was approximately 12.4 ± 1.2, 11.3 ± 0.8, and 10.6 ± 1.1 μg, respectively. The average *invA* threshold cycle (*C_T_*) values for 100, 10, 1, 0.1, and 0.01 ng DNA were approximately 11.6, 15.3, 18.7, 22.3, and 25.4, respectively, while the *invA C_T_* values of DNA samples from inoculated apples stored at 25°C for 50 days and 4°C for 110 days were 12.12 and 12.88, respectively (data not shown). Based on the above-discussed percentage of viable (green-fluorescing) cells in the total population of Salmonella on dried apples, the population of live Salmonella on dried apples after 50 days at 25°C and 110 days at 4°C was estimated to be 5.01 × 10^8^ and 3.98 × 10^8^ cells/fragment, respectively. Therefore, an estimated 31.7 and 25.2% of the total population of Salmonella inoculated on the dried apples (1.58 × 10^9^ CFU/fragment) were viable after storage for 50 days at 25°C and 110 days at 4°C, even though no colonies were recovered on agar plates at these time points.

### Assessing the role of high sugar concentration on the survival of Salmonella.

**FIG 4:**
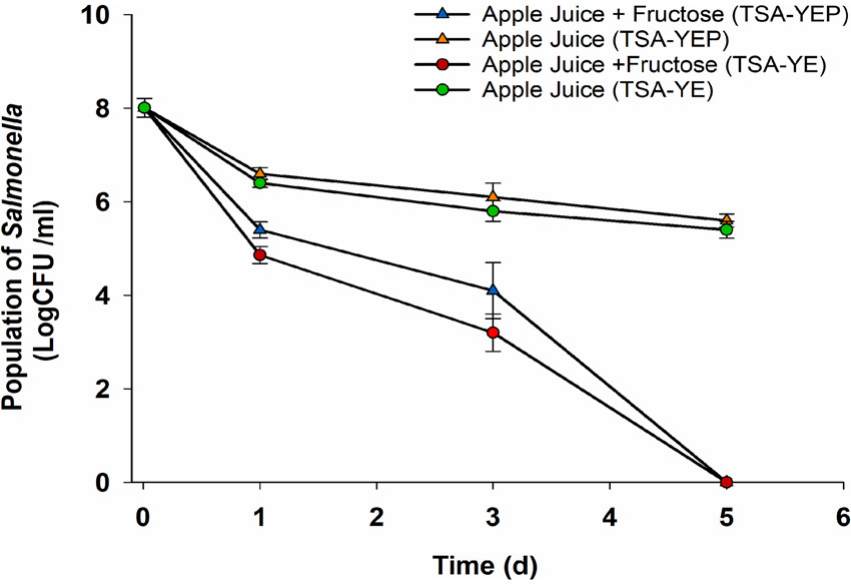
Survival of Salmonella in apple juice. Apple juice with (blue and red) or without (orange and green) fructose was inoculated with Salmonella as described in Materials and Methods and stored in the dark at 25°C. The population of Salmonella immediately after inoculation and periodically during storage was enumerated on TSA-YE (green and red) and TSA-YEP (blue and orange) as described in Materials and Methods. Dashed horizontal lines denote the limit of detection of 10 CFU/ml. The experiment was done in three independent trials, and error bars represent standard deviation.

In addition to low *a*_w_ and pH, dried apples also have quite a high sugar content, estimated to be 57% (33). To determine the effect of high sugar concentration on Salmonella survival on dried apples, we assessed survival in pasteurized apple juice with or without fructose supplementation (30%). The *a*_w_ and pH of the apple juice was 0.99 and 3.9, respectively, and the addition of fructose reduced the *a*_w_ to 0.86 while the pH was unchanged. Aerobic bacteria were not detected before inoculation (<10 CFU/ml). In apple juice without fructose, the population of Salmonella was reduced from 8 log CFU/ml upon inoculation to 6.5 log CFU/ml after 24 h at 25°C and further reduced to 5.2 log CFU/ml after 4 additional days of storage (Fig. 4). Population reductions were significantly (*P *< 0.001) higher in apple juice supplemented with 30% fructose, as the population decreased to 5.3 log CFU/ml after 24 h and was undetectable by culture at 5 days at 25°C (<10 CFU/ml) (Fig. 4). However, despite the apparent severe impact of fructose content on the recovery of Salmonella on growth media, fluorescence-based viability was not noticeably impacted. Most (92% ± 4%) of the Salmonella cells in apple juice after 5 days of storage fluoresced green, with or without added fructose (Fig. 2I and J).

## DISCUSSION

The exact routes and levels of contamination of low-moisture foods with Salmonella remain largely unknown. A high inoculum level was used in this study to inoculate dried fruit in order to better monitor population reductions, since Salmonella tends to survive longer in low-moisture environments when at a high inoculum level (29, 34). Such high bacterial populations were necessary due to the high (>10^7^ CFU/ml) detection limit for the *in situ* assessment of bacterial viability using fluorophores SYTO 9 and propidium iodide (PI) with laser scanning confocal microscopy (35, 36). In addition, we wished to compare the survival data from the current study to transcriptional analyses of inoculated dried fruit and other LMFs, which will be presented separately and where similarly high inoculum levels were used to obtain adequate RNA yields.

The rapid decline in the culturability of Salmonella bulk-inoculated on dried fruit even at the high inoculation level used in this current study was in pronounced contrast to the prolonged survival of several Salmonella strains noted upon bulk-inoculation of a different LMF, i.e., in-shell pistachios, using the same conditions and methods for inoculum preparation as employed here (37). The rapid loss of culturability in the current study emphasized the level of stress imposed by dried fruit on Salmonella. Moreover, preadapting Salmonella to an acidic pH of 4.4 before inoculating dried apples did not improve survival, as both preadapted and nonpreadapted cells of Salmonella were not detected on bulk-inoculated dried apples after the 7-day drying period at 25°C. Since rapid reductions in the levels of Salmonella were also noted with apples dried in-house, especially Granny Smith, it is unlikely that the rapid reduction on the supplied apples was due to sulfur dioxide or other antimicrobial agents that might have been added to the apples by the industry supplier. Communications from the industry supplier subsequently indicated that the dried fruits were indeed not treated with sulfur dioxide (B. Larrick, personal communication).

A previous study reported a rapid reduction in the population of Salmonella inoculated onto dried fruit (dried cranberries, raisins, dried strawberries, date paste), although the observed population reductions were lower compared to the current study (29). In the latter study, the inoculum was a suspension of a 5-strain Salmonella cocktail, preadapted to pH 4.7 and mist-inoculated at approximately 7 log CFU/g followed by drying for 1 h and storage at 4 and 25°C (29). The investigators reported reductions of 1 and 2 log CFU/g on raisins and dried strawberries, respectively, after the 1-h drying period, and by 3.5 and 4.3 log CFU/g, respectively, after storage at 25°C for 6 days (29). Differences with the current study may be due to the different inoculation methods and drying times used. Differences in strains and types of dried fruit could also result in variation in survival. Among the five strains used in the cocktails, only Salmonella enterica serovar Tennessee strain K4643 was shared between that study and the current one.

Our data suggest that the failure to recover Salmonella on growth media from the surface of dried fruit, may not be due totally to cell death but may result from the induction of the VBNC state in at least a portion of the population. This was estimated in spot-inoculated apples to correspond to 31.7 and 25.2% of the total population inoculated after storage for 50 days at 25°C and 110 days at 4°C, respectively. This ability of Salmonella to enter the VBNC state on dried fruit raises potential food safety concerns, as the pathogen would not be detected with standard culture-based methods, resulting in false-negative results and potentially allowing contaminated products to be released to the public. The potential health risk is particularly accentuated by the fact that dried fruit are considered a ready-to-eat product and are often consumed without further treatment.

The method of inoculation noticeably impacted the rate at which the VBNC state was induced in dried apples. Salmonella on spot-inoculated dried apples remained culturable much longer compared to bulk-inoculated dried apples. The faster reduction in culturability on bulk-inoculated dried apples may be due to the extended drying time (7 days) required to restore the *a*_w_ to that of uninoculated dried apples. Drying of bulk-inoculated apples inside desiccators, in contrast to air-dried spot-inoculated apples, may have conferred additional stress, thereby inducing the VBNC state sooner. Additionally, the higher cell density in the spot-inoculated dried apple, leading to the closer proximity of cells and possible quorum-sensing-related responses may have contributed to the slower induction of the VBNC state compared to the bulk-inoculated dried apples (38).

A pronounced impact of temperature was also noted, with Salmonella surviving significantly longer (*P* < 0.0001) on spot-inoculated dried apples stored at 4°C than at 25°C. This protective effect of low temperature agrees with previous studies (29, 34, 39). However, our findings suggest that the induction of the VBNC state was possible even under low temperature storage.

Entry into the VBNC state is induced in Salmonella when exposed to stresses, but the exact triggering factors in dried fruits remain unknown. Several stressors, including low pH and *a*_w_, high sugar content, aspects of the desiccation process, and naturally occurring antimicrobials are present in most types of dried fruit (29). We tested the survival of Salmonella in apple juice with or without added fructose in order to assess the impact of high sugar concentration on the induction of the VBNC state in Salmonella in the absence of a very low *a*_w_ (*a*_w_ > 0.85 in both). The recovery of Salmonella on agar media was significantly impacted by the presence of sugar, with a high sugar concentration resulting in a rapid loss of culturability, although viability was not impacted. Therefore, the induction of the VBNC state in Salmonella contaminating the dried fruit may be the result of a combination of effects, i.e., high solute concentration, low pH, and other stressors on dried fruits, and not solely low *a*_w_ or acid stress. Several tools have been used to detect bacteria in the VBNC state (40). A criterion distinguishing live and dead bacterial cells is the integrity of the cell membrane, which is expected to be intact in viable cells but compromised after cell death (41). The LIVE/DEAD *Bac*Light bacterial viability stain has been widely used to determine the viability of Salmonella (8, 13). The viability stains SYTO 9 and propidium iodide (PI) bind to nucleic acids, with PI being able to penetrate only cells with damaged cytoplasmic membranes, while SYTO 9 penetrates cells with both intact and damaged membranes (36). However, the nucleic acid-binding affinity of PI is 30-fold higher than that of SYTO 9, and thus PI outcompetes SYTO 9 in dead cells, which therefore stain red (36). The proportion of VBNC Salmonella cells on spot-inoculated dried apples stored at 25°C significantly (*P *< 0.05) decreased from 68% ± 6% at 50 days to 54% ± 4% at 110 days, suggesting that a fraction of VBNC Salmonella died upon continued storage on dried apples.

The potential of VBNC Salmonella to cause illness is poorly understood, but a growing body of evidence suggests that VBNC cells can be resuscitated under favorable conditions (11). Supplementation of growth media with pyruvate, siderophores, catalase, the dissolved oxygen-removing agent Oxyrase, and a heat-stable enterobacterial autoinducer and coculturing with eukaryotic host cells are some of the methods that have been used to resuscitate VBNC Salmonella (9, 38, 42–44). In the current study, growth media containing pyruvate yielded higher numbers of Salmonella than growth media without the supplementation. However, once CFU could no longer be detected on agar media, neither supplementation of the growth medium with sodium pyruvate nor incubation under anaerobic or microaerobic conditions appeared to promote resuscitation of VBNC Salmonella in the current study. It is worth noting that in previous studies that reported the resuscitation of VBNC Salmonella, only a fraction of the VBNC population was resuscitated while a significant proportion remained unculturable (8, 9, 44). The mechanisms involved in the resuscitation of VBNC cells remain poorly understood, but the bacterial agent involved, the stressors that trigger the VBNC state and their duration of exposure, as well as the age of the VBNC cells appear to play important roles in resuscitation (11, 45). Therefore, although procedures used in the current study to resuscitate VBNC Salmonella were not successful, other factors that VBNC Salmonella may encounter in the environment, food processing plants, or in the human gastrointestinal tract may allow for resuscitation.

In conclusion, the findings from this study showed that Salmonella can enter the VBNC state on dried fruit, which could lead to an underestimation of the viable cells of Salmonella using culture-based microbiological methods. Further studies are needed to understand the key environmental factors that trigger the induction and resuscitation of the VBNC state in Salmonella and the molecular mechanisms that are involved. Additionally, further research needs to be done on the physiology and virulence of VBNC Salmonella. Lastly, future technological advances that permit lower limits of detection of VBNC cells are expected to enable investigations at low inoculum levels that would more adequately simulate natural contamination events. Such studies will further our understanding of the practical and public health implications of the induction and persistence of the Salmonella VBNC state in dried fruit.

## MATERIALS AND METHODS

### Characteristics of dried fruit and measurement of water activity (*a*_w_), pH, and Brix.

Dried apples were provided directly by an industry cooperator as bulk-packed dried-fruit fragments with average dimensions of 9 by 9 by 2 mm and an average weight of 0.2 g; whole dried strawberries and raisins were provided by the same industry cooperator as entire dried fruit, with average weights of 3 and 0.7 g per individual fruit, respectively. For in-house preparation of dried apples, fresh Red Delicious and Granny Smith apples were purchased from local stores, peeled, sliced into fragments (10 by 10 by 2 mm) and dehydrated for 12 h using a 6-tray Cabela household dehydrator (model 54-1646; Sidney, NE, USA). They were then stored in Whirl-Pak stomacher bags (750 ml; Nasco, Fort Atkinson, WI) until use.

The *a*_w_ of dried fruit before and after inoculation, after drying, and periodically during storage was determined using a benchtop water activity meter (AquaLab model 3TE; Decagon Devices, Pullman, WA). The *a*_w_ of samples inoculated with Salmonella could not be measured due to pathogen concerns for the benchtop water activity meter. Therefore, dried fruits that were inoculated with heat-killed nonpathogenic Escherichia coli K-12 at the same volume/weight as dried fruit inoculated with Salmonella were used instead. Dried strawberries were sliced into smaller pieces in order to fit the loading chamber of the water activity meter, while dried apples fragments and raisins were analyzed without size reduction. To determine the pH of dried fruit homogenates, the dried fruit (10 g) was macerated in 10 ml distilled water (dH_2_O) in 50-ml conical centrifuge tubes (VWR Int.) using a flame-sterilized spatula and then vortexed for 60 s. The pH of the homogenate was determined using a benchtop pH meter (Orion model 410Aplus; Thermo Scientific, Waltham, MA). The amount of dissolved solids (including sugars) in rinsates of dried fruit was measured as Brix (°Bx). Dried fruit (5 g) was transferred into 10 ml dH_2_O in 50-ml conical centrifuge tubes (VWR Int.), the mixtures were vortexed at high speed for 2 min, and the Brix of the rinsates was determine using a pocket refractometer PAL-3 (Atago, Minato-ku, Tokyo, Japan). The *a*_w_, pH, and Brix were determined in triplicate for each type of dried fruit.

### Bacterial cultures and inoculum preparation.

The Salmonella enterica strains used in this study are shown in Table 2 and were kindly provided by Nathan Anderson (U.S. Food and Drug Administration, Institute for Food Safety and Health, Bedford Park, Illinois). The strains were selected because of their involvement in LMF-associated outbreaks and also because they have been employed in storage and thermal resistance studies (46). Media and conditions routinely used for growth include Trypticase soy broth (TSB) (Becton, Dickinson & Co., Sparks, MD) containing 0.6% yeast extract (TSB-YE) (Becton, Dickinson & Co.) and TSB-YE supplemented with 1.2% agar (TSA-YE) (Becton, Dickinson & Co.) incubated statically at 37°C for 24 h. Bacterial culture stocks were preserved in TSB containing 20% glycerol (Fisher Scientific, Fair Lawn, NJ) at −80°C. To prepare the inoculum suspension, frozen stock cultures of individual strains were streaked on TSA-YE plates and incubated at 37°C for 24 h. A single colony was then inoculated in 3 ml TSB-YE in 15-ml conical centrifuge tubes (Becton, Dickinson & Co.), incubated overnight at 37°C, and 100 μl of each culture was spread-plated on TSA-YE followed by incubation at 37°C for 24 h to achieve a bacterial lawn. For each strain, the bacterial lawn from two agar plates was harvested using 10-μl sterile disposable plastic loops (Fisher Scientific), resuspended in 10 ml of sterile deionized water (dH_2_O), washed twice, and then combined in equal volumes to achieve a 5-strain cocktail (50 ml) used to inoculate the LMFs.

**TABLE 2:** Salmonella enterica strains used in this study

Strain	Source and reference
Salmonella Agona strain 447867	Rice and wheat puff cereal outbreak, 2008 (51)
Salmonella Tennessee strain K4643	Peanut butter outbreak, 2006–2007 (52)
Salmonella Montevideo strain 488275	Black pepper outbreak, 2009–2010 (53)
Salmonella Mbandaka 698538	Sesame tahini paste outbreak, 2013 (54)
Salmonella Reading	Unknown source

### Bulk-inoculation, storage, and microbiological analysis of dried fruit.

Before inoculation, 5 g of each type of dried fruit was transferred into 10 ml of dH_2_O in 15-ml centrifuge tubes (VWR Int., Radnor, PA). The mixtures were vortexed at high speed, serially diluted in dH_2_O, and appropriate dilutions were plated on TSA-YE and xylose lysine deoxycholate (XLD) agar (Becton, Dickinson & Co.), followed by incubation at 37°C for 24 h to enumerate total aerobic bacteria and Salmonella, respectively. Each inoculation was performed in triplicate. To inoculate the dried fruit, 200 g of each type of dried fruit was weighed into separate sterile Whirl-Pak stomacher bags (750 ml; Nasco, Fort Atkinson, WI) and bulk-inoculated at 10% volume of cell suspension per weight by transferring 20 ml of cell suspension into the bags in a series of four additions of 5 ml each, with each addition followed by vigorous shaking by hand for 30 s. After inoculation, the bags were shaken again for 30 s to further mix the content, and the inoculated dried fruit was spread on perforated ceramic plates inside a Nalgene polypropylene desiccator (Thermo Scientific, Waltham, MA) containing Drierite (W.A. Hammond Drierite Co. Ltd., Xenia, OH) as the desiccant. The desiccators were kept inside an incubator at 25°C and 33 to 48% rH for 7 days at which time the *a*_w_ of the inoculated dried fruit was within 98% to 120% of uninoculated products. To enumerate Salmonella on dried fruit immediately after inoculation and after drying, 5 g of dried fruit was transferred into 10 ml of dH_2_O inside 50-ml conical centrifuge tubes (VWR Int.). The mixtures were vigorously shaken at high speed for 60 s, serially diluted in dH_2_O, and appropriate dilutions were plated on TSA-YE and XLD agar plates, followed by incubation at 37°C for 24 h. The differences between Salmonella CFU on TSA-YE and the selective medium XLD would correspond to potentially injured cells that can be recovered on the nonselective medium TSA-YE but fail to grow on XLD (47). The experiment was performed in three independent trials, with duplicate samples analyzed at each time.

Further assessment of survival of Salmonella on dried apples utilized Salmonella preadapted to low pH as described (29). Individual strains of Salmonella enterica serovar Agona, *S*. Tennessee, Salmonella enterica serovar Montevideo, Salmonella enterica serovar Mbandaka, and Salmonella enterica serovar Reading were grown in 1 ml TSB-YE at 37°C for 24 h. From the overnight bacterial cultures, a 100-μl cell suspension of each strain was individually spread-plated onto TSA-YE supplemented with 1% glucose (TSA-YEG), followed by incubation at 37°C for 24 h. Harvesting of the cells from the lawns and preparation of the 5-strain cocktail as inoculum were done as described previously. The inoculated dried apples were dried as described previously for 7 days, and enumerations were done as described previously on TSA-YE and XLD agar plates. When indicated, the plates were also incubated at 37°C for 24 h microaerobically using a Bactrox hypoxic chamber (SHEL LAB, Cornelius, OR; model 388L) set at 5% O_2_, 10% CO_2_, complemented with nitrogen, and anaerobically using an anaerobic chamber (COY, Grass Lake, MI) maintained at 5% CO_2_, 10% H_2_, and 85% N_2_.

### Enrichment for Salmonella contaminating dried fruit.

When Salmonella contaminating dried fruit was below the limit of detection (20 CFU/g), samples were enriched according to the FDA Bacteriological Analytical Manual (48) protocol with modifications. Briefly, 5 g of the dried apples, dried strawberries, and raisins was transferred into 15 ml of lactose broth (Becton, Dickinson & Co.) in 50-ml centrifuge tubes (VWR Int.) and incubated at 37°C for 24 h. After 24 h, 100 μl of the lactose broth was transferred into 9 ml Rappaport-Vassiliadis broth (Oxoid, Basingstoke, UK) and incubated at 42°C for 48 h. Suspensions (100 μl) from the lactose and Rappaport-Vassiliadis broths were plated on XLD agar plates, which were incubated at 37°C for 24 h.

### Spot-inoculation and cold storage of dried apples.

Fragments of the industry-supplied dried apples were placed in sterile Petri dishes and spot-inoculated with 20 μl per fragment (approximately 10^9^ CFU) of the 5-strain Salmonella strain cocktail described above. The inoculated fragments were air-dried inside a biosafety cabinet for 3 h with the Petri dish lid off, and then the Petri dishes were covered, sealed with parafilm, and stored in the dark at 25 or 4°C. For enumerations, individual fragments were transferred into 1 ml of dH_2_O in 1.5-ml Eppendorf tubes, and the population of Salmonella was enumerated on TSA-YE as described previously. Dilutions were also plated on TSA-YE with 0.1% sodium pyruvate (TSA-YEP) (Acros Organics, Fair Lawn, NJ, USA) and incubated at 37°C for 24 h. Spot-inoculation of dried apples was employed in three independent trials, with duplicate samples for each time point.

### Survival of Salmonella in apple juice.

Organic pasteurized apple juice was purchased from a local store and stored at 4°C until usage. The pH, Brix, and background microbiota of the juice was determined as described previously. Fructose (9 g; Sigma-Aldrich, St. Louis, MO) was added to 30 ml of apple juice at 30% wt/vol to increase the Brix of the juice to that of the dried apple rinsate described above. To test the effect of high solute concentration on the survival of Salmonella, 30 ml of apple juice with or without fructose in 50-ml conical centrifuge tubes (VWR Int.) was inoculated with the 5-strain Salmonella cocktail described above at 10^8^ CFU/ml and stored in the dark at 25°C. Immediately after inoculation and periodically during storage, 1 ml of inoculated apple juice with or without fructose was serially diluted in sterile dH_2_O, and appropriate dilutions were spread-plated on TSA-YE and TSA-YEP. The experiment was repeated in three independent trials with duplicate samples at each time point.

### Viability staining and visualization of Salmonella on dried fruit.

Dried fruits were bulk-inoculated with Salmonella and dried to the original *a*_w_ for 7 days as described previously. Rinsates from the inoculated fruit were stained with the LIVE/DEAD BacLight bacterial viability kit (Invitrogen, Eugene, Oregon) according to the manufacturer’s instructions. Briefly, the green (SYTO 9) and red (propidium iodide) stains were mixed in equal volumes, and 1 μl of the mixture was added to 300 μl of the rinsate from the inoculated dried fruit and incubated in the dark at 25°C for 10 min. The suspensions were then spotted (3 μl) onto glass slides, dried under ambient conditions for 2 min to fix the cells onto the slides, and visualized with a fluorescence microscope (BX63; Olympus, Waltham, MA). Green fluorescence (live bacteria) was captured with fluorescein isothiocyanate (FITC) filters, while Texas Red (TR) filters were used to detect red fluorescence (dead bacteria). The cell suspension used as inoculum and rinsates of uninoculated dried fruit were similarly stained and visualized. Visualization of Salmonella in the rinsates from dried fruit was performed in three independent trials with duplicate samples processed per trial. Images were acquired from at least three different microscopy fields per replicate of each trial using Olympus cellSens image analysis software (Olympus, Waltham, MA). Fluorescing (live and dead) bacterial cells were counted with ImageJ (49) to compute the percentage of viable cells as the number of green fluorescing cells over the total number of cells. VBNC Salmonella in inoculated apple juice with or without fructose was detected by staining 300 μl of apple juice as described for inoculum suspensions. Visualization was performed using 2 μl of stained apple juice, and viable cells were enumerated as described. The experiment was performed in three independent trials.

Laser scanning confocal microscopy was used to visualize Salmonella on the surfaces of spot-inoculated dried apples after drying for 3 h, at 50 and 110 days of storage at 25°C, and at 110 days of storage at 4°C. Briefly, an equal-volume mixture of SYTO 9 and PI stains was diluted with dH_2_O 1:4, and 5 μl was spotted on the inoculated spot, followed by incubation in the dark at 25°C for 15 min. A small fragment (3 by 3 mm) with a flat surface was excised from the stained region using a flame-sterilized razor blade and placed in 300 μl dH_2_O in a glass cell culture dish (P35G-1.5-20-C; MatTek, MA, USA) with the stained surface immersed in the water. Image acquisition was performed using a Zeiss LSM 880 confocal laser scanning microscope (Carl Zeiss Inc. Thornwood, NY, USA) at the Cellular and Molecular Imaging Facility at North Carolina State University. A multiline argon laser light source and an inverted LD C-Apochromat 40×/1.1 W Korr M27 water immersion objective lens were used for all experiments. Simultaneous acquisition with two main dichroic beam splitters (MBS) at 488 and 561 nm was used to image the double marker line with the pinhole maintained at 1 Airy unit (AU) (1.1 μm). SYTO 9 was excited at 488 nm and emission was collected at 499 to 561 nm, while PI was excited at 561 nm with emission collected at 578 to 669 nm. Images were processed using the Zeiss Zen Blue software (Zeiss, Obercohen, Germany). The experiment was conducted in three independent trials with duplicate samples in each trial, and image acquisition from at least three different microscopy fields per replicate of each trial. Fluorescing bacterial cells were enumerated as described previously.

### Bacterial DNA extraction and quantitative real-time PCR.

Individual fragments of spot-inoculated dried apples after drying for 3 h and at 50 and 110 days of storage at 25 and 4°C, respectively, were transferred into 1 ml of dH_2_O in 1.5-ml microcentrifuge tubes (VWR Int.). The mixtures were vortexed at high speed for 60 s, the apple fragments were removed, and the remaining suspensions were centrifuged at 15,000 × g and 25°C for 2 min. Genomic DNA (gDNA) of the bacterial cells in the resulting pellet was extracted with the Qiagen DNeasy blood and tissue kit (Qiagen Sciences Inc., Germantown, MD) according to the manufacturer’s instructions. DNA was extracted in duplicate in two independent trials, and DNA concentration was determined with NanoDrop spectrophotometer (Model ND2000C; Thermo Scientific).

The quantity of the target gene *invA* in each gDNA sample was determined using the StepOnePlus real-time PCR system (Applied Biosystems, Foster City, CA). The real-time PCR mix consisted of 1× iTaq Universal SYBR green supermix (Bio-Rad Laboratories Inc, Des Plaines, IL), 600 nM (each) forward (*invA*Forward, 5′-TCGCCAGTACGATATTCAGTG-3′) and reverse (*invA*Reverse, 5′-AGGCCTTCGGGTTGTAAAGT-3′) primers, and the gDNA template in a 20-μl reaction mixture. The thermocycler conditions used included 95°C for 10 min followed by 40 cycles of 95°C for 15 s and 60°C for 1 min. For dried apples after 3 h of drying, five reactions containing 0.01, 0.1, 1, 10, and 100 ng of template gDNA were run. DNA samples from 50 and 110 days of storage at 25 and 4°C, respectively, were used as template in separate reactions. The threshold cycle (*C_T_*) values and concentrations were exported into Microsoft Excel 365 (Redmond, WA) and a plot of average *C_T_* values against gDNA concentration was computed. Since *invA* is a single-copy gene in Salmonella (50), the copies of *invA* detected are expected to correspond to the population of Salmonella (live and dead) in the sample. Total population of Salmonella/fragment was calculated from the standard curve generated with gDNA from dried apples after 3 h of drying, with number of viable cells.

$$\left(\text{cells}/\text{fragment}\right)=\frac{N_{\text{v}}}{N_{\text{T}}}\times T_{\text{c}}$$

where *N*_v_ and *N*_T_ are fluorescence microscopy-based counts of viable (green) cells and total (green or red) cells, respectively, determined as described previously, while *T*_c_ is the total population of Salmonella (live and dead) calculated from the qPCR data.
